# Building resilience in animal research: a two-year mixed methods survey of organizational support programs

**DOI:** 10.3389/fvets.2025.1736974

**Published:** 2026-02-03

**Authors:** Lauren Young, Sally Thompson-Iritani, Rita U. Bellanca, Fabienne Ferrara, Kirsten Bell, Tara Martin, Megan R. LaFollette

**Affiliations:** 1 The 3Rs Collaborative, Denver, CO, United States; 2 Office of Research, University of Washington, Seattle, WA, United States; 3 Washington National Primate Research Center, University of Washington, Seattle, WA, United States; 4 ConScienceTrain (Consulting and Training in Science), Berlin, Germany; 5 Animal Sciences & Technologies, AstraZeneca, Waltham, MA, United States; 6 Refinement and Enrichment Advancements Laboratory, Unit for Laboratory Animal Medicine, University of Michigan, Ann Arbor, MI, United States

**Keywords:** animal research, compassion fatigue, culture of care, job satisfaction, professional quality of life, resilience, employee retention, organizational wellness programs

## Abstract

**Introduction:**

People who work with research animals often experience both meaning and stress from their careers. It can be rewarding to care for animals and contribute to science, yet also challenging to euthanize animals, see them in distress, and work in a stigmatized, fast-paced field. Organizations conducting animal research increasingly recognize this and are implementing programs to support employee wellness. This longitudinal project describes the implementation and evaluation of compassion fatigue resiliency programs over 2 years.

**Methods:**

Five organizations participated in a pre-post longitudinal trial implementing institutional compassion fatigue resiliency programs. Participants were evaluated before the intervention and 2 years later with a mixed methods online survey designed to evaluate professional quality of life, job satisfaction, and retention. Quantitative data were analyzed via general linear models and qualitative data were analyzed thematically.

**Results:**

Fifty-two participants responded to both surveys. From baseline to year two, there were no significant changes in professional quality of life, job retention, or job satisfaction. However, participant understanding and implementation of strategies to combat compassion fatigue increased. Additionally, professional quality of life remained associated with job satisfaction and retention (*p* < 0.05). In free-response text, participants frequently mention animal-research related factors (62%) and organizational culture (48%) as factors that make compassion fatigue worse. Across the years, participants were more likely to mention mental health-related factors (9% at baseline to 34% at year two) and less likely to mention animal-research (54–32%) as making compassion fatigue better.

**Discussion:**

This is the first longitudinal survey assessing the impacts of organizational compassion fatigue programs on professional quality of life. Although there were no statistical differences in job satisfaction and retention across time, there continued to be a link between professional quality of life, job satisfaction, and retention. Furthermore, participants did increase understanding and implementation of strategies to combat compassion fatigue. Participants indicated that their wellness was impacted by organizational culture, animal research, and mental health factors. Considering positive qualitative feedback on our program and the link between retention and professional quality of life, our results suggest there may be workplace benefits to promoting a culture of care and supporting resiliency.

## 1 Introduction

Working with animals in research settings, contributing to the development and safeguarding of a healthy life for humans and animals, can be meaningful and fulfilling. Employees in this field have enabled significant scientific breakthroughs and medical advancements, for both human and veterinary diseases – while also supporting strong animal welfare. However, this work can also be challenging, both physically and emotionally. For example, research personnel can develop attachments to their animals which ultimately leads to an emotional toll when these animals experience stress or pain or are ultimately euthanized (1–4). Beyond the unique factors of animal research, time pressure, workload, and interpersonal dynamics can have a negative impact on employee wellbeing (5–8). Overall, these ongoing stressors can lead to sustained negative effects on personnel such as the development of compassion fatigue, which is comprised of burnout and secondary traumatic stress (9).

As poor workplace wellness and compassion fatigue have a multitude of negative impacts, attempts should be made by institutions to promote employee well-being and a culture of care (7, 10). Preliminary research indicates that employee retention and job satisfaction may be supported by promoting compassion satisfaction, reducing burnout, and developing a positive organizational culture (7). For example, workplace wellbeing programs may include open dialogue that recognizes the emotional impact of the work, stress management or individual coping strategies, as well as attempts to address systemic conditions, such as workload, team communication, leadership practices, and ethical reflection (7, 8, 10–12).

Current research in this area is typically review based or cross-sectional, focusing on the factors that contribute to compassion fatigue, resilience, and how programs can be developed to reduce the risk of compassion fatigue (3, 6, 11). Therefore, there is a gap in the long-term evaluation of compassion fatigue resiliency programs for personnel that work with animals in research. This project’s objective was to assist organizations with the implementation of a compassion fatigue resiliency program and conduct a longitudinal evaluation of such a program across 2 years. Our specific aims were to quantify the relationship between professional quality of life, job satisfaction, and job retention over time. Based on previous research, we hypothesized that after implementation of an organizational compassion fatigue resiliency program that professional quality of life, job satisfaction, and job retention would improve. Regardless of our results, we hoped to provide proof of concept, resources, and an initial step to rigorous evaluation of institutional compassion fatigue resiliency programs.

## 2 Materials and methods

All procedures and waived signed consent protocols were approved by University of Michigan’s Human Research Protection Program Institutional Review Board (IRB), protocol # HUM00207730. Participants provided waived signed consent via a yes or no question on the online survey platform after reading an informed consent document. No IACUC approval was sought as there were no interactions between the researchers and animals specifically to support the survey.

### 2.1 Institutions

In 2021, direct email and verbal communication was used to recruit institutions to the study. Verbal communication included both presentations and newsletter emails by members of the 3Rs Collaborative. Inclusion criteria for institutions included: being located in the USA or Canada, not currently having a compassion fatigue resiliency program or having a newly established program and being willing to work with the 3Rs Collaborative to implement a program and recruit participants.

Ultimately, five institutions met inclusion criteria and participated in the study across the years. This included three research institutes, and two large pharmaceutical companies. Each institution contained a possible sample size between 27 and 429 individuals. Altogether they represented approximately 703 eligible participants.

### 2.2 Participants

At each institution, one to three employees were identified to coordinate study participation. These individuals were typically directors, managers, or supervisors with authority to coordinate compassion fatigue resiliency activities. For the baseline survey, participants were recruited, and data was collected between February 11 and March 22, 2022. For the year 2 survey, participants were recruited and data was collected between February 20 and March 29, 2024. At baseline, eligible employees at each institution were invited to participate via three separate emails and a physical flyer hung within the workplace. In year 2, eligible employees were asked to take the follow-up survey via three additional emails. Inclusion criteria for participants were: being over the age of 18 and currently working at one of the included institutions; there were no exclusion criteria.

Participants read an informed consent document that assured them that their responses would be kept confidential (including from supervisors) and then affirmed documentation of their waived signed consent. They then completed an online questionnaire estimated to take an average of 10 min via Qualtrics. Participants were informed that they could skip any question that made them feel uncomfortable. Although 3 of the authors had access to email addresses that could identify individual participants during data collection, during the data analysis phase the responses were de-identified with a participant and institutional code to ensure they were kept anonymous and confidential. Potentially identifiable information was only accessible to core research team members.

### 2.3 Program

**Figure 1:**
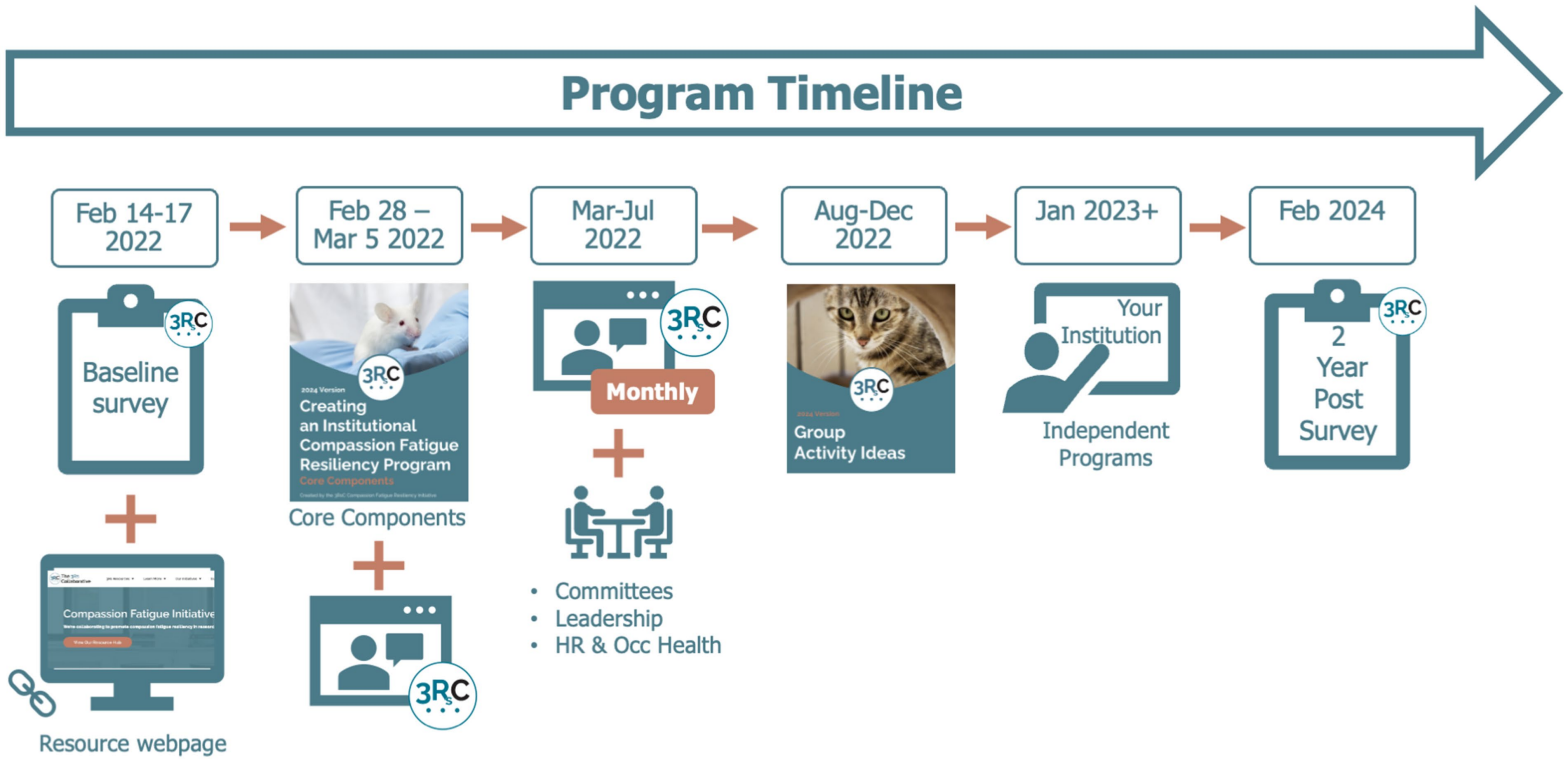
Timeline of survey and compassion fatigue program. This figure shows the timeline that surveys were administered in relation to when tools and information were provided to the participating organizations. Surveys were administered at baseline and 2 years post baseline. Resources were provided intensely for the first 6 months by 3RsC, and then institutions were encouraged to develop their own activities.

Figure 1 details the timing of the surveys and interventions. After the baseline survey from February to March 2022, a detailed packet with core information on launching a program was provided to program leads, and organizations were sent physical posters to hang in visible areas. From March to July of 2022, monthly webinars hosted by the 3Rs Collaborative were provided to the participating organizations. These organizations were also encouraged to set up monthly committee meetings. After this time, organizations were encouraged to continue to host independent activities for their staff until the final survey in February of 2024. They were given additional materials to support these efforts such as group activity ideas, a culture of care-specific packet, a copy of the survey, and educational reading materials to distribute to employees. In January of 2023 participating institutions were given revised materials based on project findings from year one including a packet on a manager’s role in compassion fatigue resiliency and suggestions for peer-to-peer resources. All resources from this project can be found online via the 3Rs Collaborative’s compassion fatigue resiliency resource hub^1^.

### 2.4 Measures

A mixed-method survey was developed by the 3Rs Collaborative’s Compassion Fatigue Resiliency committee. The survey questions were developed via a review of the relevant literature in combination with expert opinions from those in survey methodology and laboratory animal science fields. Questions were developed using validated survey tools (e.g., professional quality of life scale (9)) survey tools adapted to the topic of compassion fatigue (e.g., modified nurse retention index (13)), or tools created for the purpose of the survey. Scales created for the purpose of the survey were reviewed by the initiative, piloted, and revised as necessary.

Overall, participants were asked 78–86 questions each year. For personnel that worked hands-on with animals, additional questions were asked to determine if retention was unique for these types of roles. Questions were subdivided into subsections as described below. All survey text and scoring can be found in the Supplementary Table S1.

#### 2.4.1 Demographics and work factors

Participants were first asked to complete a waiver of signed consent, followed by their age for inclusion purposes. They were then asked for their email to allow responses to be linked between baseline and year 2 surveys. Participants were then asked work and demographic questions including working role, years of work in the field, gender, average hours of work in a week, highest education and finally whether or not they work hands-on with research animals. Participants were also asked to rate the degree of stress/pain that the majority of animals experience in their care based off the official United States Department of Agriculture (USDA) pain and distress categories for laboratory animal research (14) [previously shown to impact professional quality of life (3)].

#### 2.4.2 Professional quality of life knowledge and experiences

To evaluate professional quality of life, participants were asked to rate their compassion fatigue via a descriptive scale from one to five and via the 30-question professional quality of life scale [PROQOL, (9)]. They were also asked about their familiarity with the definition of compassion fatigue, effective strategies to combat compassion fatigue resiliency, and whether they have implemented strategies to combat or experienced compassion fatigue in the past. Finally, they were asked free response questions as to what makes their compassion fatigue worse or better.

#### 2.4.3 Job satisfaction and retention

To evaluate job satisfaction, participants were asked the seven item Brief Index of Affective Job Satisfaction Scale (15) which results in a score of 1 to 20 from low to high job satisfaction. To evaluate retention, participants were asked the modified nurse retention index (13) where “nursing” was replaced with “research animals” which results in a score of 6 to 48 from low to high anticipated retention. Participants that previously reported working hands-on with research animals were then specifically asked about retention using the same modified scale but with the word “hands-on” added.

#### 2.4.4 Resiliency and perceived stress

To evaluate resiliency, participants were asked the CDRISC-2 (Connor Davidson Resiliency Scale) (16) which includes two items resulting in a summed score of 0 to 8. They were also asked about their levels of perceived stress via the Perceived Stress Scale (17). This scale included ten items rated 0 to 4, which were ultimately reversed to result in a score of 0 to 40 from low to high perceived stress.

#### 2.4.5 Program effectiveness

To evaluate program participation and effectiveness, participants were first asked which 3RsC developed activities they participated in, followed by what they thought was the most beneficial aspect of a compassion fatigue program. Participants were also asked if they had any suggestions to improve the 3RsC’s or their own institution’s program, and if they had any further comments about their current wellness and subsequent suggestions for the implementation of a program. Finally, participants were asked how likely they are to recommend the 3RsC compassion fatigue resiliency program to a friend or colleague using the net promoter score (NPS) scale (18).

### 2.5 Data analysis

#### 2.5.1 Participant inclusion

Of the approximately 723 potentially eligible participants at baseline, 198 individuals completed the initial baseline survey with enough data to be included in our baseline publication (7). Of those, 53 individuals also completed responses with enough data to analyze in year 2. Therefore, our overall response rate was approximately 7% while our longitudinal response rate from baseline to year 2 was approximately 26%. Ultimately each institution had 7 to 18 participants in the longitudinal dataset.

#### 2.5.2 Quantitative analysis

Data analysis was performed in R Version 2025.09.02. Quantitative data were analyzed and are presented with both descriptive statistics and general linear models. Continuous data are presented as mean and standard deviation (SD). Counts are presented as *n* and percent (%). Duplicate responses were identified via matching email addresses, and the most complete, or recent response if all were equally complete, was retained. Only participants that rated their level of compassion fatigue were included (i.e., those who only answered demographic data were excluded). For use in general linear models, categories with less than 10 responses were collapsed into larger buckets. Summary scales were calculated according to instructions for each individual scale.

Three types of general linear mixed models were run with different outcome variables. The first type included either job satisfaction or job retention as the dependent variables and burnout, compassion satisfaction, and secondary traumatic stress as independent variables. The second type included either burnout, compassion satisfaction or secondary traumatic stress as the dependent variables and job satisfaction and job retention as independent variables. A third type of model was run that included either resiliency or perceived stress as the dependent variable and burnout, compassion satisfaction and secondary traumatic stress as independent variables to control for these factors. All models additionally included work factors (animal stress/pain, hands-on work, role, years of work, and hours per week), and demographics (highest education, age, sex). Participant nested in institution was included as a random blocking factor. Finally, survey timepoint (baseline vs. year 2) was included as an independent variable.

Significance level was set at *p* < 0.05 and independent variables including years in the field, role, age, gender, highest education were removed from the final model if they did not meet this cut off. Results are presented as means +/− standard deviation.

This representative analysis was used: Job Retention = Timepoint + Compassion Satisfaction + Burnout + Secondary Traumatic Stress + Animal Stress/Pain + Hands-On Work with Animals + Role + Years + Hours per Week + Highest Education + Sex + Participant (Institution).

#### 2.5.3 Qualitative analysis

Open-ended questions were assessed using a coding manual developed during baseline qualitative analysis [see (7)]. The baseline manual was developed using inductive, bottom-up, content analysis to derive themes from all respondent answers. See (7) for complete information on the development of the coding manual. The same manual was used for qualitative data beyond baseline to allow for effective comparison of responses from baseline to subsequent survey timepoints.

Coding was performed by LEY and inter-rater reliability was assessed by having an additional individual (AS), who was not involved in the manual creation process, code a random 20% of the data.

Longitudinal qualitative results were analyzed and grouped by the question asked and qualitatively compared between baseline and year 2 survey result to tease apart the valence of responses and corresponding thematic codes. This was done by taking the number of participants whose response was coded with a particular theme/subtheme, divided by the total participants that responded to the survey. Our team determined that a change in at least 10 respondent answers (approximately 20% of our total sample), between baseline and year 2, was considered qualitatively important for interpretation.

## 3 Results

### 3.1 Study participants

A total of 53 participants completed enough of the survey at both timepoints to be included in this longitudinal project. Table 1 contains complete demographics for the longitudinal study participants from the final year of survey data. Participants worked primarily as researchers and animal caretakers, although several other job roles were represented. Participants were from either a research institute or a pharmaceutical organization and primarily held graduate, veterinary, or bachelor’s degrees as their highest level of education. Participants primarily identified as female and most worked 40 h a week. Close to half of participants had been working with research animals from 10–19 years. The majority of participants worked hands-on with research animals. Finally, close to half of participants reported that the animals they care for experience ‘minor stress or pain of a short duration’. Descriptive statistics of outcome measures are presented in Table 2.

**Table 1:** Participant demographic and work information (*N* = 53).

Variable	Baseline		Year 2	
	*N*	% of Total	*N*	% of Total
Role				
Animal caretaker	12	23%	6	11%
Researcher	16	30%	16	30%
Manager	5	9%	5	9%
Research technician	4	8%	4	8%
Veterinarian	4	8%	4	8%
Veterinary Technician	2	4%	2	4%
IACUC/Ethical Review	8	15%	6	11%
Other	2	4%	9	17%
Highest education				
Graduate or Veterinary Degree	22	42%	22	42%
Bachelor’s Degree	21	40%	21	40%
Associate’s Degree	6	11%	6	11%
High School Diploma	4	8%	4	8%
Institution type				
Research Institute	29	55%	27	51%
Pharmaceutical Organization	26	49%	24	45%
Academic	0	0%	0	0%
Sex				
Female	42	79%	41	77%
Male	11	21%	11	21%
Transfemale or Transmale	0	0%	0	0%
Prefer Not to Answer	0	0%	1	2%
Animals stress/pain				
Little or no discomfort or stress	7	13%	8	15%
Minor stress or pain of a short duration	22	42%	25	47%
Moderate stress or pain of a short duration	20	38%	16	30%
Procedures which cause severe pain near, at, or above the pain tolerance threshold of unanesthetized conscious animals	2	4%	2	4%
During an average week, about how many hours do you work				
<40	6	11%	5	9%
40	27	51%	30	57%
41–49	15	28%	16	30%
50+	5	9%	2	4%
How many years have you worked with research animals?				
<10	17	32%	16	30%
10–19	28	53%	23	43%
20+	8	15%	14	26%
Do you work hands on with research animals?				
Yes	40	75%	39	74%
No	13	25%	14	26%

**Table 2:** Descriptive statistics for professional quality of life, retention, job satisfaction, resiliency (CDRISC), and perceived stress scale.

Descriptive scale	*N* (participants)	Baseline		Year 2	
		Mean	Standard Deviation	Mean	Standard Deviation
Compassion satisfaction	49	36.74	10.440	36.75	9.5816
Burnout	49	21.60	7.461	23.02	7.6145
Secondary traumatic stress	49	20.57	7.632	20.92	7.8352
General retention index	48	30.96	4.218	30.16	4.6626
Hands-on retention index	35	30.30	3.951	27.77	7.3011
Job satisfaction	48	35.49	8.692	35.84	6.0045
CDRISC	49	6.41	1.553	6.16	1.280
Perceived stress scale	49	20.02	4.323	19.90	4.501

### 3.2 Professional quality of life knowledge and experiences over time

**Figure 2:**
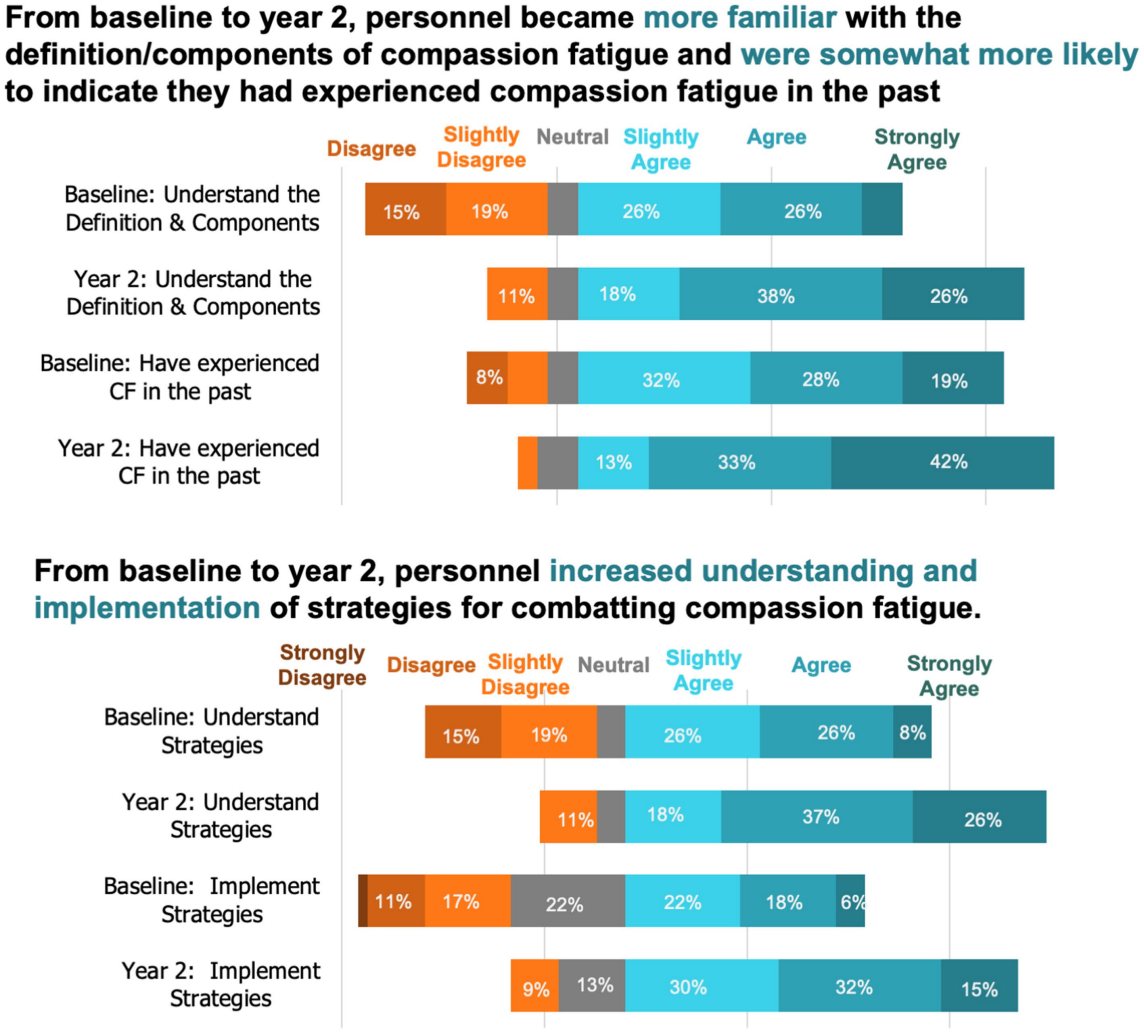
Animal research personnel’s experiences and understanding of compassion fatigue (*N* = 53).

Research animal personnel were asked about their compassion fatigue knowledge and experiences at both timepoints (Figure 2, Table S3). Most participants agreed that they were familiar with the definition and components of compassion fatigue and that they had experienced compassion fatigue in the past at both baseline and year. In year 2, more participants agreed they now understood effective strategies for combatting compassion fatigue and had implemented strategies to combat compassion fatigue.

**Figure 3:**
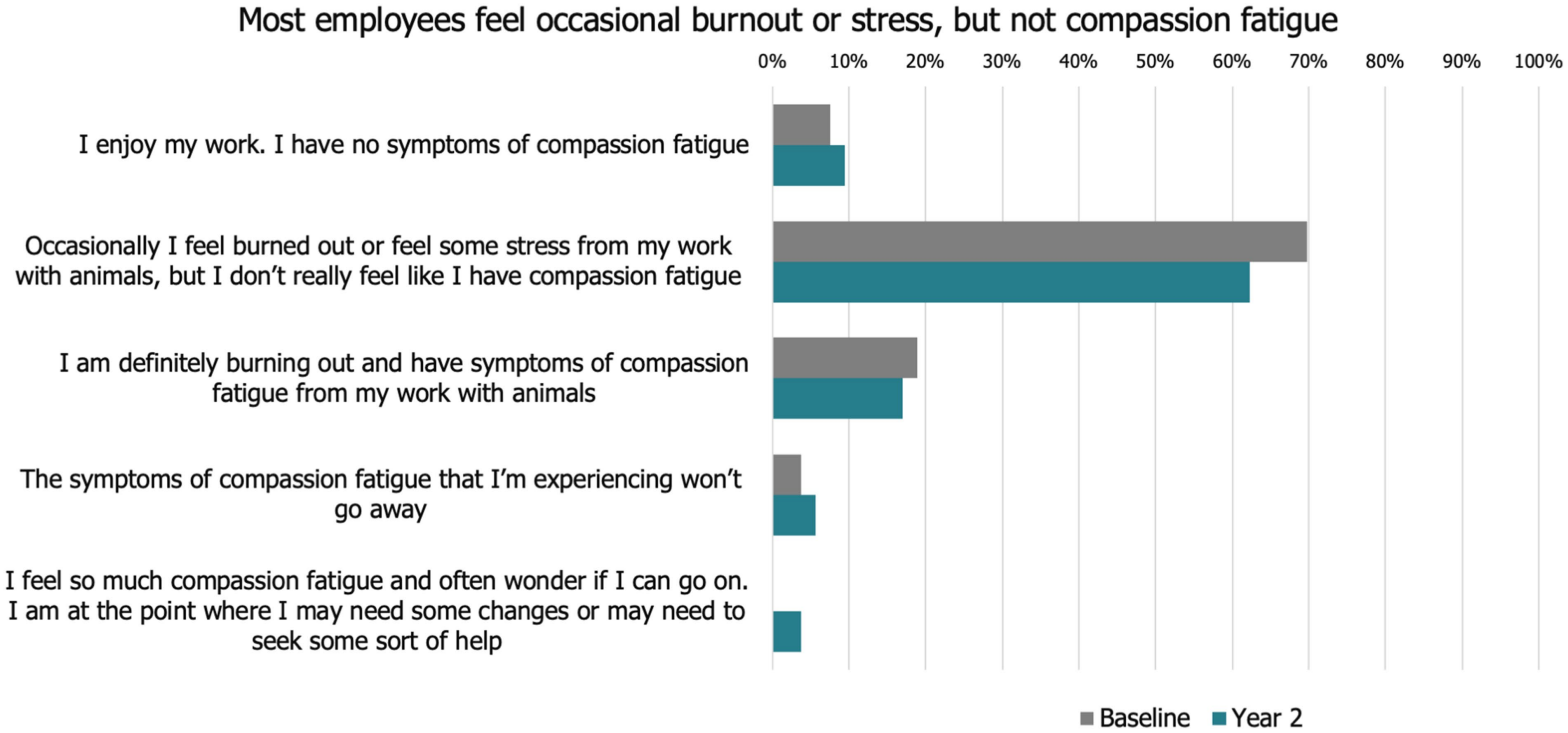
Descriptive compassion fatigue scale from baseline to year 2.

**Figure 4:**
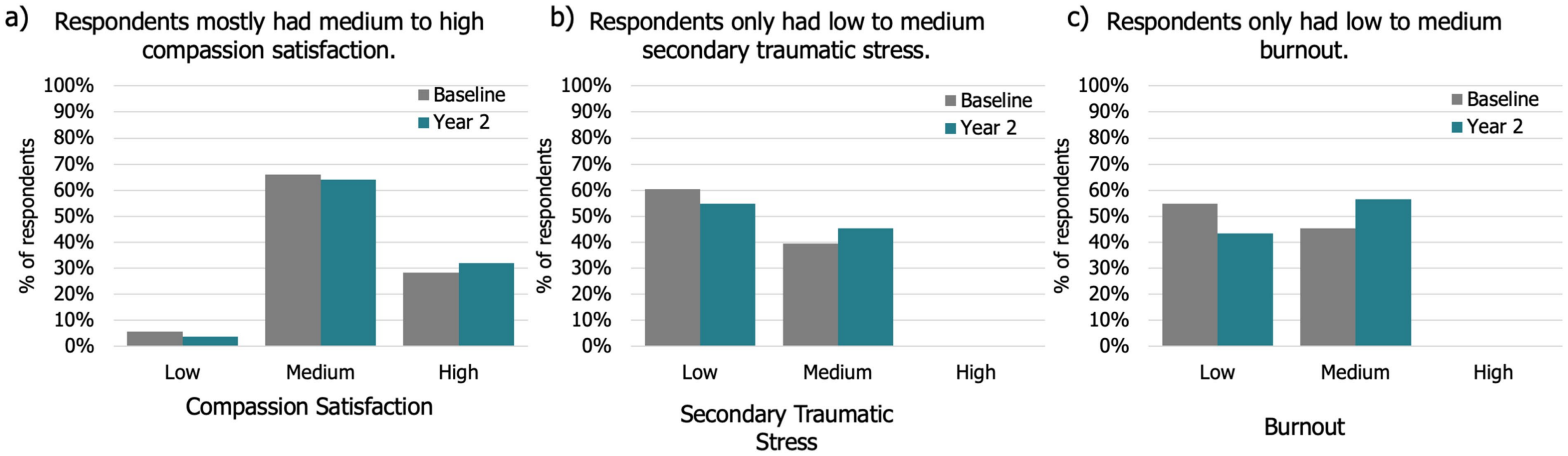
Comparison of PROQOL factors from baseline to year two: **(a)** Compassion satisfaction, **(b)** burnout, and **(c)** secondary traumatic stress.

Overall, from baseline to year 2 most participants occasionally felt burnt out or stressed, but did not feel like they had compassion fatigue, which was supported by their responses to the PROQOL scale (Figures 3, 4a–c, Table S3). Descriptively there was no major change from year to year in these metrics.

### 3.3 The link between workplace wellness, job satisfaction, and retention

In this study, job satisfaction was significantly impacted by compassion satisfaction, secondary traumatic stress and burnout, while retention was impacted by compassion satisfaction (Tables 3, 4). Specifically, personnel who reported higher levels of job satisfaction also reported higher levels of compassion satisfaction and lower levels of secondary traumatic stress, burnout, and animal stress/pain. Personnel had higher compassion satisfaction also had stronger anticipated job retention both overall and for hands-on animal work. Higher retention for hands-on work with research animals was also associated with higher degree levels. The impact of compassion satisfaction had a large effect size, while all other effect sizes were medium.

**Table 3:** Associations between job retention and satisfaction.

Independent variables	Job satisfaction	Retention	Hands on retention
Timepoint	*F*_1,43_ = 0.51, *p* = 0.4791	*F*_1,49_ = 1.06, *p* = 0.3088	*F*_1,63_ = 2.05, *p* = 0.1576
Professional quality of life			
Compassion satisfaction	***F***_**1,74**_ **= 20.91, *p* < 0.0001 (+), η**^**2**^**ₚ = 0.22**	***F***_**1,86**_ **= 22.59, *p* < 0.0001 (+), η**^**2**^**ₚ = 0.21**	***F***_**1,63**_ **= 15.83, *p* = 0.0002 (+), η**^**2**^**ₚ = 0.20**
Burnout	***F***_**1,72**_ **= 8.83, *p* = 0.0040 (−), η**^**2**^**ₚ = 0.11**	F_1,85_ = 0.68, *p* = 0.4123	F_1,63_ = 0.28, *p* = 0.5976
Secondary traumatic stress	***F***_**1,87**_ **= 5.72, *p* = 0.0189 (−), η**^**2**^**ₚ = 0.06**	*F*_1,85_ = 0.002, *p* = 0.9610	*F*_1,63_ = 0.18, *p* = 0.6749
Work factors			
Animal stress/pain	***F***_**1,82**_ **= 8.41, *p* = 0.0048 (−), η**^**2**^**ₚ = 0.09**	*F*_1,82_ = 0.32, *p* = 0.5752	*F*_1,63_ = 1.08, *p* = 0.3032
Hands on work	*F*_1,49_ = 1.46, *p* = 0.2328	*F*_1,48_ = 0.02, *p* = 0.8967	–
Demographics			
Hours per week	*F*_1,77_ = 2.07, *p* = 0.1547	*F*_1,79_ = 0.27, *p* = 0.6052	*F*_1,63_ = 2.72, *p* = 0.1039
Age	–	–	–
Sex	–	–	–
Years in role	–	–	–
Job role	–	–	*F*_1,63_ = 1.74, *p* = 0.1839
Highest education	–	**–**	***F***_**1,63**_ **= 6.57, *p* = 0.0128** Graduate/Vet = higher retention**, η**^**2**^**ₚ = 0.09**

The associations from three general linear models of research animal personnel’s job satisfaction and retention. Job satisfaction was measured via the affective job satisfaction scale (*n* = 48). Job retention was evaluated both for working with research animals generally (*n* = 48) and in hands-on role (*n* = 35) by a modified nurse retention scale. Participants were asked to complete the professional quality of life questionnaire about work factors (animal stress/pain, whether they worked hands-on, and their institution). They were also asked the hours they worked per week, years working in the field, job role, age, sex, and highest education. F = F-statistic. (+) The independent variable has a positive association with the dependent variable. (−) The independent variable has a negative association with the dependent variable. η^2^ₚ = partial η^2^ (effect size) for significant results. Bold indicates a significant result.

**Table 4:** The associations from three general linear models of research animal personnel’s compassion satisfaction, burnout and secondary traumatic stress.

Independent variables	Compassion satisfaction	Burnout	Secondary traumatic stress
Timepoint	*F*_1,47_ = 0.53, *p* = 0.4711	*F*_1,43_ = 0.68, *p* = 0.4149	*F*_1,42_ = 0.26, *p* = 0.6154
Job retention			
Job satisfaction	***F***_**1,89**_ **= 52.20, p = <0.0001 (+), η**^**2**^**ₚ = 0.37**	***F***_**1,81**_ **= 29.00, *p* < 0.0001 (−), η**^**2**^**ₚ = 0.26**	*F*_1,71_ = 2.14, *p* = 0.1479
Retention	***F***_**1,85**_ **= 17.57, *p* = 0.0001 (+), η**^**2**^**ₚ = 0.17**	***F***_**1,68**_ **= 4.38, *p* = 0.0401 (−), η**^**2**^**ₚ = 0.06**	***F***_**1,56**_ **= 4.30, *p* = 0.0427 (−), η**^**2**^**ₚ = 0.07**
Work factors			
Animal stress/pain	*F*_1,87_ = 3.00, *p* = 0.0865	*F*_1,82_ = 3.57, *p* = 0.7639	*F*_1,73_ = 0.39, *p* = 0.5351
Hands on work	*F*_1,50_ = 0.01, *p* = 0.9300	*F*_1,55_ = 0.09, *p* = 0.0114	*F*_1,62_ = 0.01, *p* = 0.9223
Demographics			
Age	–	–	
Sex	–	*F*_1,55_ = 2.57, *p* = 0.0536	***F***_**1,51**_ **= 3.64, *p* = 0.0333** (F > M)**, η**^**2**^**ₚ = 0.07**
Hours per week	*F*_1,87_ = 0.08, *p* = 0.7760	***F***_**1,79**_ **= 6.71, *p* = 0.0114 (+), η**^**2**^**ₚ = 0.08**	–
Years in role	**–**	–	–
Job role	–	–	–
Highest Education	–	–	–

Compassion satisfaction, burnout and secondary traumatic stress were measured via a professional quality of life scale (*n* = 49). Participants were asked to complete a job satisfaction scale, a modified nursing retention scale, and about work factors (animal stress/pain, whether they worked hands-on, and their institution). They were also asked the hours they worked per week, years working in the field, job role, age, sex, and highest education. F = F-statistic. (+) The independent variable has a positive association with the dependent variable. (−) The independent variable has a negative association with the dependent variable. Bold indicates a significant result.

In turn, self-reported compassion satisfaction was significantly impacted by job satisfaction and job retention (Tables 3, 4). Personnel who reported increased compassion satisfaction indicated increased job satisfaction and retention, with a large effect size. Self-reported burnout was significantly impacted by job satisfaction (with a large effect size), job retention, and hours worked per week (the latter two with medium effect sizes). Personnel who reported increased burnout indicated decreased job satisfaction and retention and were more likely to report working more than 40 h each week, with medium effect sizes. Finally, personnel who reported increased secondary traumatic stress also indicated decreased job retention and were more likely to be females than males, both with a medium effect size.

### 3.4 Assessment of resiliency and perceived stress over time

Research animal personnel resiliency was significantly impacted by compassion satisfaction (Table 5). Personnel who reported higher levels of resiliency also reported higher levels of compassion satisfaction. None of the other investigated factors significantly impacted resiliency, and none of the investigated factors significantly impacted personnels’ perceived stress (Table 5).

**Table 5:** Associations between resiliency, perceived stress, and professional quality of life.

**Independent Variables**	**Resiliency**	**Perceived Stress**
Timepoint	F_1,46_ = 2.20, p=0.1447	F_1,43_ = 0.19, p=0.6632
**Professional Quality of Life**		
Compassion Satisfaction	**F**_**1,84**_ **= 14.15, p<0.0003 (+)**	F_1,82_ = 0.95, p=0.3331
Burnout	F_1,77_ = 0.07, p=0.7930	F_1,64_ = 2.91, p=0.0928
Secondary Traumatic Stress	F_1,82_ = 2.49, p=0.1187	F_1,83_ = 0.69, p=0.4076
**Work Factors**		
Animal Stress/Pain	F_1,81_ **=** 0.06, p=0.8080	F_1,88_ = 2.93, p=0.0908
Hands On Work	F_1,47_ = 3.00, p=0.0897	F_1,48_ = 0.26, p=0.6103
**Demographics**		
Hours per week	F_1,77_ = 2.07, p=0.1547	F_1,83_ = 0.47, p=0.4929
Age	–	–
Sex	–	–
Years in role	–	–
Job role	–	–
Highest Education	–	**–**

The associations form two general linear models of participants resiliency and perceived stress with professional quality of life, work factors, and demographics. Bold indicates a significant effect. F = F-statistic. (+) = the independent variable has a positive association with the dependent variable.

When asked, *“what makes your compassion fatigue worse?,”* respondents mention all themes roughly the same amount at both baseline and year 2, with animal research-specific factors and organizational culture factors mentioned the most frequently (Figure 4a). Animal research-specific factors include aspects related directly to research animals such as euthanasia, specific procedures and translatability or perceived importance of studies. As one participant describes, “Compassion fatigue is most front-and-center for me in scenarios where there is a higher-than-normal frequency of invasive procedures or euthanasia, [or] when I feel like there is not a strong justification for why the study must be done.” Negative organizational culture factors include poor work-life balance, unpleasant interactions with staff, and not feeling valued. As one participant describes, “Feeling powerless to drive big change in a big program, feeling like I cannot make a difference for animals, [a] lack of receptivity by any animal users/caretakers to change [and] being contacted outside of work to address work questions.” Mental health-related factors and compassion fatigue-specific factors were mentioned fewer times by longitudinal participants when asked what makes compassion fatigue worse.

When asked, *“what makes your compassion fatigue better?,”* respondents most frequently mentioned organizational culture (Figure 1). Furthermore, from baseline to year 1, respondents mentioned mental health-related factors more, and animal research-specific factors less (Figure 4b). Factors relating to a positive organizational culture were cited as making compassion fatigue better for participants at both baseline and year 2. For example, one participant states “High morale on the team, [and] if my direct reports are feeling satisfied with their work and position, makes my compassion fatigue symptoms better.” Others mention “taking a break walking in between timepoints,” “teamwork” and “compassion from management and peers.” Animal research-specific factors are mentioned less frequently at year. Participants mention “good survival rate of surgery,” “positive reinforcement [training]” and “seeing researchers get meaningful results and show that they are considering the animals in their study designs.” Mental health-related factors are mentioned more frequently at year 2 than baseline, such as “exercising and laughing,” “being around others and socializing” and “getting enough sleep.”

Finally, when asked, *“what is most beneficial for your compassion fatigue?,”* respondents mention all themes roughly the same amount at these timepoints, with compassion fatigue-specific factors mentioned most frequently (Figure 4c). When participants discuss compassion-fatigue specific factors they mention compassion fatigue resources stating, “the materials gave us ideas and confidence about how to move our program forward.” They also mention normalizing compassion fatigue and that a “better understanding and the realization that compassion fatigue and satisfaction are real” and that even just “knowing someone is studying this phenomenon” are beneficial.

When individuals were directly asked, *“how does the culture of your workplace impact your quality of life?,”* 13 (39.40%) respondents mentioned positive aspects of their workplace culture. These responses included statements such as, “leadership understands compassion fatigue and ways to minimize it are built into the work schedules in an effort to reduce symptoms” and “there is a culture of care and appreciation for the research personnel in my workplace that is positive.” Alternately, 16 (48.49%) mentioned negative aspects of their workplace culture. These responses included statements such as, “I find that a negative culture makes feelings of compassion fatigue worse. Also, if the culture does not acknowledge these feelings and expects me to be a robot, it significantly increases feelings of compassion fatigue” and “the culture surrounding our work is that we have deadlines to meet and need to satisfy our managers.” Finally, 4 (12.12%) mentioned both positive and negative aspects, or their responses were considered neutral.

## 4 Discussion

To our knowledge, this is the first published longitudinal investigation of professional quality of life in research animal personnel after implementation of institutional compassion fatigue resiliency programs. The project was run as a pre-post intervention trial in which five partner organizations across the United States were given free resources to support their staff. Although results may not be widely generalizable due to limited sample, this work is an important proof of concept and initial step to rigorous evaluation of institutional compassion fatigue resiliency programs.

In this study, we created numerous resources to support institutional compassion fatigue programs and distributed them among our participating institutions. These resources can be accessed from the 3Rs Collaborative’s website compassion fatigue resource hub (see footnote 1). During this program, we found that Although, there were no statistically significant changes in professional quality of life over the 2 years of evaluation, participants’ understanding of compassion fatigue and strategies to combat it increased over the two years, as did their implementation of strategies to combat compassion fatigue. Furthermore, we found that throughout the years, compassion satisfaction and compassion fatigue (e.g., secondary traumatic stress and burnout) were associated with employee retention and job satisfaction. Additionally, open-ended feedback and overall rating of the program with its developed resources was positive. Finally, in free response answers, participants emphasized that organizational culture, animal research factors, and mental health factors can all affect their compassion fatigue in either positive or negative ways.

## 3.5 Program utilization and effectiveness

Participants reported engaging in the following components of the 3RsC compassion fatigue program in decreasing order: webinar on an overview of CFR (57%), webinar on culture of care (42%), enrichment activities (42%), webinar on mindfulness (38%), in person activities (34%), accessing reading materials on compassion fatigue (32%), poster viewing (26%), accessing mindfulness or gratitude materials (21%), webinar on communication & trust in the workshop (19%), accessing independent activities (19%), memorial activities (13%), and webinar on meaning making (9%). We note that institutions provided additional independent activities outside of this list that were not evaluated as they varied between locations.

When asked how likely participants were to recommend the 3RsC compassion fatigue resiliency program to a friend or colleague, there was overall a positive net promoter score of +14.6 (Promoters = 35.4%, Passives = 43.8%, Detractors = 20.8%). Promoters select 9 or 10, indicating an enthusiastic recommendation. Passives select 7 or 8, indicating a satisfied but unenthusiastic endorsement. Detractors select 6 or lower, indicating a dissatisfaction with the program. On average, participants were classified as “Passives” (mean score = 7.63+/− 1.96).

## 3.6 Qualitative results

**Figure 5:**
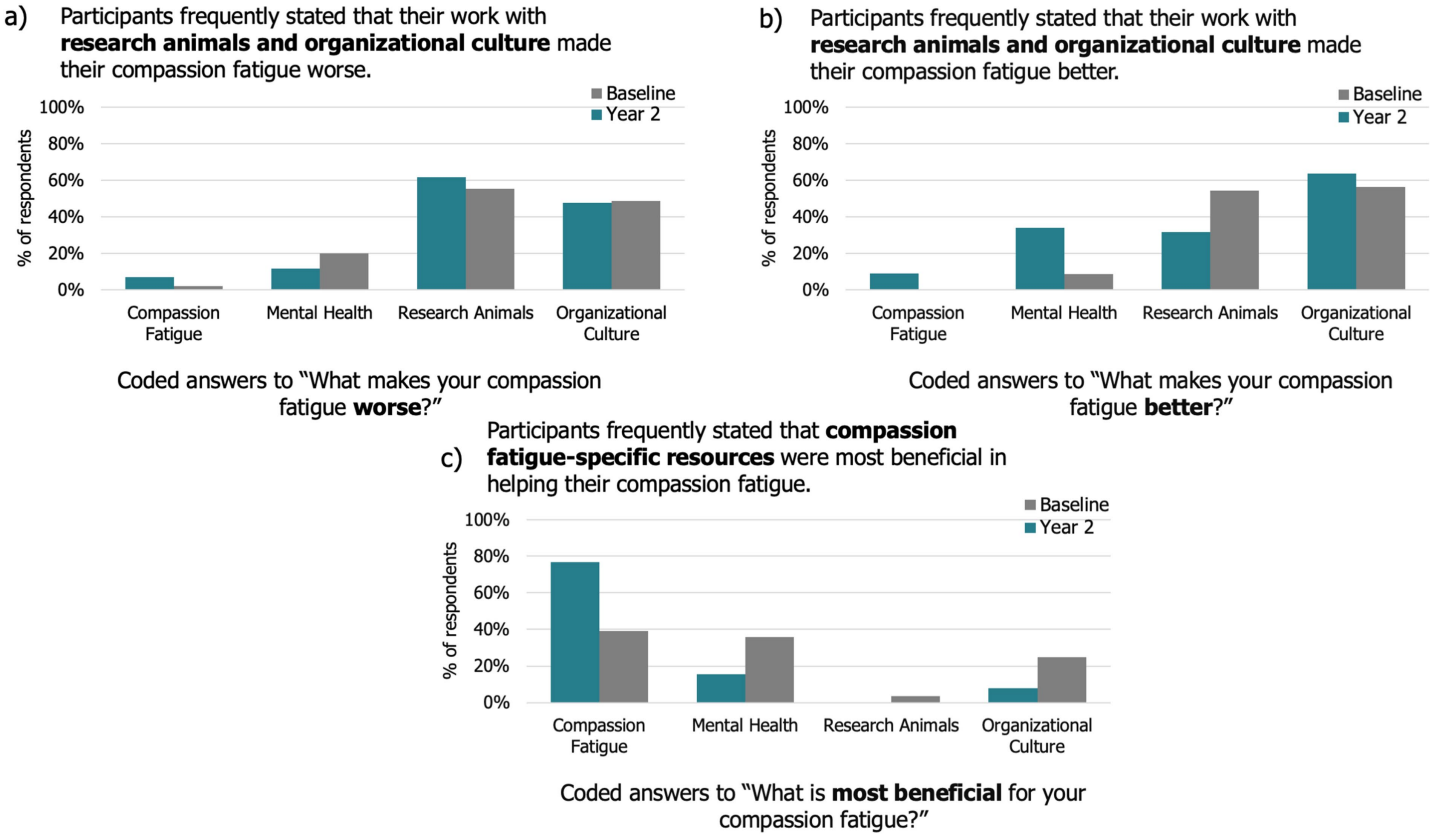
Key factors that impact compassion fatigue in animal research personnel (*n* = 53). The percentage of research animal personnel whose responses to each question included each theme at baseline and year 2: **(a)** “What makes your compassion fatigue worse?” Respondents felt that animal research-specific factors and organizational culture factors make their compassion fatigue worse; **(b)** “What makes your compassion fatigue better?” Respondents felt that mental health-related factors make their compassion fatigue better; **(c)** “What factors are most beneficial for your compassion fatigue?” Respondents felt that compassion fatigue-specific factors are most beneficial in helping their compassion fatigue.

Of the 53 participants included in the longitudinal study, 42 (79%) responded to “what makes your compassion fatigue worse”; 44 (83%), responded to “what makes your compassion fatigue better”; 13 (25%) responded to “what would be most beneficial about a compassion resiliency program” and 34 (64%) responded to “how does the culture of your workplace impact your professional quality of life.” A detailed summary of qualitative results, and comparisons between baseline and year 2, can be found in in Figures 5a–c and Supplementary Table S2.

### 4.1 Professional quality of life is consistently linked to job satisfaction and retention

In this study, professional quality of life (e.g., compassion satisfaction and compassion fatigue which is comprised of burnout and secondary traumatic stress) was linked to job satisfaction and retention at both baseline and year 2. Compassion satisfaction was positively associated with job satisfaction and retention, while burnout was negatively associated with job satisfaction. Finally, resiliency was positively associated with compassion satisfaction. This provides additional support for the likely importance of a “culture of care” at research organizations which includes caring for animals, people, science, and openness (8, 12, 19). Programs that support research employee’s professional quality of life are therefore likely good not only for the individual and the animal (3), but also the workplace itself.

In this study, there was no effect of pre/post implementation on professional quality of life, job satisfaction, retention, resiliency or perceived stress. These factors neither increased nor decreased over the two-year study period. There are several possibilities for this outcome. Although it is possible that the program simply had no effect on employees, our targeted qualitative data does not seem to support this explanation. Instead, we suspect these results could be related to this project’s timing and educational interventions. At the start of this project in January of 2022, it was early “post-COVID” during which time vaccines were widely available and major shutdowns stopped but work and travel loads had not fully rebounded from the global pause in 2020. By the project end in 2024 work and travel load were largely often “back to normal.” Therefore, there may have been a confounding factor of increased workplace stress by the end of the project. Additionally, giving individuals training on the topic of compassion fatigue may have allowed them to recognize it in themselves and be more comfortable discussing it at the end of the project. Indeed, in year 1 more personnel indicated they were more familiar with the definition and components of compassion fatigue. As this project did not include a control group without an intervention it is impossible to determine a potential time effect. Where feasible, we recommend future evaluations include a no treatment control group.

### 4.2 Organizational culture continues to contribute to professional quality of life

Our results continue to support the idea that organizational culture is a key factor influencing workplace quality of life. Factors related to organizational culture were frequently mentioned by participants in response to open-ended questions about compassion fatigue resiliency. Furthermore, when asked directly about how workplace culture impacts compassion fatigue, most participants discussed its importance in either supporting or harming quality of life. They cited important factors such as feeling valued by managers, having a voice and being listened to, and work-life balance. The resources developed as part of this project may support more a more positive institutional culture. These include a packet outlining a manger’s role in a wellness program, culture of care training, and peer-to-peer support resources. We also note that, in quantitative data, working more than 40 h a week was associated with increased burnout. Therefore, it is critical for workplaces to commit to a comprehensive culture of care including supporting staff including providing them adequate time (19), given the impacts of short staffing, stress, and working long hours highlighted by this and our previous study (7).

### 4.3 Animal-specific factors are linked to job satisfaction

Unique factors to working in research that involves animals, such as exposure to higher reported levels of animal stress and pain, were linked to job satisfaction in quantitative results and mentioned in response to open-ended questions. Again, this supports the intertwined nature of human and animal welfare (7) as well as the 3Rs. Refinement of studies, rotation of staff, and allowing staff members to have some control over their job duties such that exposure to animal suffering is reduced are likely to be beneficial to employees and the workplace (8, 12). Directly acknowledging the challenges of working in animal research appears critical to supporting staff. Numerous resources developed as part of this project can support institutions in this aim including recommendations for developing a tribute/memorial event, compassion fatigue resiliency posters, training webinars, and group activities such as creating enrichment for animals. Again these finding indicate the importance establishing a culture of care at research institutions (8, 12, 19).

### 4.4 Demographics: higher education linked to higher retention. Females may be more at risk for stress

In this study, participants with higher levels of education (e.g., graduate or veterinary degrees) compared to 4-year degrees reported higher general and hands-on job retention, despite experiencing similar levels of compassion fatigue-related factors like secondary traumatic stress and burnout. These workers may be more educated about or prepared for working with research animals or have access to other avenues of support such as professional societies [e.g., AVMA’s veterinary wellbeing resources (20)]. They may also have more insight into the translation of research which was previously cited as impactful for compassion fatigue (7) and have more of a planning role, and therefore more control over animal enrichment and their involvement in euthanasia which is associated with more positive PQOL (3). Furthermore, they themselves may play a key role in supporting other staff’s psychological well-being (21). It is also possible that their reported retention may be related to an innate passion for the field that led them to seeking a graduate or veterinary degree or the “sunk cost” of schooling.

In our analysis, female participants reported higher secondary traumatic stress than males. This is in line with other research showing that female nurses and veterinary professionals experience higher rates of compassion fatigue versus males (22–24). However, we note that our study was not designed to test gender differences with only 11 males participating.

### 4.5 Compassion fatigue and mental health resources are beneficial

Based on participant feedback, compassion fatigue and mental health resources are also important to support compassion fatigue resiliency. A dedicated compassion satisfaction program like the one implemented by our study could be a key component of supporting individuals working in animal research. From year one to two, the percentage of participants who understood the definition for compassion fatigue increased slightly, while those that understood effective strategies for combatting compassion fatigue increased by 23%. Additionally, at the end of the project 30% more participants indicated that they had implemented strategies to combat compassion fatigue. These findings were reaffirmed in free-responses where participants stated a desire for resources related to both compassion-fatigue and general mental health support.

Workplace wellness programs specialized for animal research staff allow individuals to find validation, support, education, and a non-judgmental space where they can bond with other staff members and access resources for support (8, 21). In fact, simple disclosure of psychological stressors has been found to be beneficial for compassion fatigue in other settings (25) as has acceptance and commitment therapy (26). Furthermore, daily well-being practices have been found to increase compassion satisfaction (27). Again, the resources developed as part of this project fulfill all these suggestions and therefore can be implemented by individual institutions to promote compassion fatigue resiliency.

### 4.6 Limitations and future directions

This project includes several limitations due to its design. First, as this survey was cross-sectional and did not include a control group that did not receive an intervention, it is not possible to determine the directionality or causation of the associations. Therefore, it could be possible that rather than higher professional quality of life leading to higher reported job retention that the two are simply associated without causation. Additionally, as explored earlier, the confound of time may have masked any effects of the intervention. Future evaluations on this topic would benefit from a larger sample size and longer term randomized control trial to determine causation.

Another limitation is the inability to incorporate participant program engagement into the quantitative analyses. Engagement with workplace wellness resources was not measured at baseline and activity participation beyond the 3RsC offerings was not captured. As a result, participation data were available only in year 2, were incomplete, and therefore unsuitable for inclusion in our pre-post mixed effects model. Nevertheless, 77% of participants reported implementing strategies to address compassion fatigue resiliency, suggesting substantial engagement with the program. Furthermore, individual differences may cause certain individuals to gain benefits form only certain programmatic pieces. Future research should assess engagement throughout the study period with all relevant activities which would allow future projects to evaluate both new and established programs in a controlled manner and incorporate participant engagement into quantitative analyses.

Additionally, our work is limited to individuals that chose to stay at their place of employment throughout the years and therefore does not include information from those who left the field or their company. Therefore, it is possible that our results are artificially more positive than if we could have included those individuals. Those leaving may have had lower retention, satisfaction, and quality of life or progressed up the career ladder with positive support and compassion satisfaction. Finally, this work may not be widely generalizable beyond this sample as it only includes results from 5 organizations across the United States which is not a representative sample. Despite these limitations, our project still provides important insight into the development of workplace wellness programs to improve professional quality of life in research animal personnel.

## 5 Conclusion

In conclusion, this project created resources for and demonstrated the feasibility and some positive impacts of implementing an organizational compassion fatigue resiliency program. It is a proof of concept and initial step to rigorous evaluation of such programs which can be used a template for future longitudinal, control trials. Furthermore, it provides additional evidence supporting the link between professional quality of life, job satisfaction and job retention. Additionally it furthers the finding that animal research personnel are impacted by both workplace culture and animal-research specific factors. These results provide further encouragement to organizations to use and create their own resources that improve employee professional quality of life through improving their overall culture of care. These resources should include support of employee wellbeing, commitment to the 3Rs, animal welfare, and quality science, as well as open, honest communications about research involving animals.

## Data Availability

The raw data supporting the conclusions of this article are not readily available due to privacy. Requests to access the datasets should be directed to the corresponding author. The anonymized data is provided as a Supplementary file.

## Footnotes

1
https://3rc.org/compassion-fatigue/institutional-resources/

## Supplementary material

SUPPLEMENTARY TABLE S1 Survey text and coding scheme.

SUPPLEMENTARY TABLE S2 Qualitative coding manual, results, and descriptions.

SUPPLEMENTARY TABLE S3 Descriptive data results and percentages.
